# Supplementary material for: Endoplasmic reticulum disruption stimulates nuclear membrane mechanotransduction
Source: Nat Cell Biol. 2025 Dec 9;28(1):125–34. doi: 10.1038/s41556-025-01820-9 (PMC12807876; doi:10.1038/s41556-025-01820-9)
Supplement: Supplementary file 1 — Reporting Summary [file 41556_2025_1820_MOESM1_ESM.pdf]

nature portfolio

Corresponding author(s): *Double-anonymous peer review submissions: write DAPR and your manuscript number here instead of author names.*

Last updated by author(s): Oct 23, 2025

Reporting Summary

Nature Portfolio wishes to improve the reproducibility of the work that we publish. This form provides structure for consistency and transparency in reporting. For further information on Nature Portfolio policies, see our Editorial Policies and the Editorial Policy Checklist.

Statistics

For all statistical analyses, confirm that the following items are present in the figure legend, table legend, main text, or Methods section.

n/a	Confirmed	
☐	☒	The exact sample size (*n*) for each experimental group/condition, given as a discrete number and unit of measurement
☐	☒	A statement on whether measurements were taken from distinct samples or whether the same sample was measured repeatedly
☐	☒	The statistical test(s) used AND whether they are one- or two-sided<br>*Only common tests should be described solely by name; describe more complex techniques in the Methods section.*
☒	☐	A description of all covariates tested
☐	☒	A description of any assumptions or corrections, such as tests of normality and adjustment for multiple comparisons
☐	☒	A full description of the statistical parameters including central tendency (e.g. means) or other basic estimates (e.g. regression coefficient) AND variation (e.g. standard deviation) or associated estimates of uncertainty (e.g. confidence intervals)
☐	☒	For null hypothesis testing, the test statistic (e.g. *F*, *t*, *r*) with confidence intervals, effect sizes, degrees of freedom and *P* value noted<br>*Give P values as exact values whenever suitable.*
☒	☐	For Bayesian analysis, information on the choice of priors and Markov chain Monte Carlo settings
☒	☐	For hierarchical and complex designs, identification of the appropriate level for tests and full reporting of outcomes
☐	☒	Estimates of effect sizes (e.g. Cohen's *d*, Pearson's *r*), indicating how they were calculated

Our web collection on statistics for biologists contains articles on many of the points above.

Software and code

Policy information about availability of computer code

Data collection	U2OS cell and GUV membrane confocal imaging data were collected in NIS-Elements Software (4.13.04) and NIS-Elements Software (5.21.03). Intravital confocal microscopy of zebrafish larvae were collected in NIS-Elements Software (3.22.14). Multi-photon laser imaging data of zebrafish larvae were collected in Leica LAS X 4.8.1.29271. Super Resolution imaging data of U2OS cell were collected in ZEN Black 3.0 SR FP2, v16.0.20.306. FLIP Imaging data were acquired in Leica LAS X 3.5.7.23225.
Data analysis	FLIP imaging data were processed in LAS X 3.5.7.23225. segmented in FIJI (ImageJ) and analyzed in custom Python3 scripts (https://github.com/zazadovv/ER_Nucleus.git). Structured-illumination microscopy (SIM) reconstruction was performed in ZEN 3.0 SR FP2 (16.0.20.306 black). Segmentation of SIM masks were performed in Imaris v10.2 before visualizing in custom written python3 scripts (https://github.com/zazadovv/ER_Nucleus.git). Zebrafish image stacks were deconvolved and denoised using default method and denoise.ai tools in Nikon NIS elements (5.21.03). Multiphoton imaging results were processed using Leica LAS X, 4.8.1.29271. All live, permeabilized cell and GUV membrane imaging experiments were prepossessed, segmented and analyzed in custom Python3 scripts (Nuclear membrane analysis: https://github.com/joeshen123/Nuclear-Membrane-Binding-4 Analysis.git, ER membrane analysis: https://github.com/joeshen123/ER-Structure-Analysis.git , GUV membrane binding analysis: https://github.com/joeshen123/GUV-Protein-Binding-Analysis-Program.git).GUV equilibrium binding isotherm was calculated and plotted using Curve Fitting Box (V 25.1) from Matlab R2025a before imported and modified in Adobe AI. Representative images were prepared in FIJI (ImageJ) and plots were generated with seaborn or matplotlib from Python 3 as vector graphs and subsequently modified in Adobe AI.

For manuscripts utilizing custom algorithms or software that are central to the research but not yet described in published literature, software must be made available to editors and reviewers. We strongly encourage code deposition in a community repository (e.g. GitHub). See the Nature Portfolio guidelines for submitting code & software for further information.

Data

Policy information about availability of data

All manuscripts must include a data availability statement. This statement should provide the following information, where applicable:

- Accession codes, unique identifiers, or web links for publicly available datasets
- A description of any restrictions on data availability
- For clinical datasets or third party data, please ensure that the statement adheres to our policy

Numerical source data supporting the findings of this study are provided with the publication. Additional data are available from the corresponding author(s) upon reasonable request.

Research involving human participants, their data, or biological material

Policy information about studies with human participants or human data. See also policy information about sex, gender (identity/presentation), and sexual orientation and race, ethnicity and racism.

Reporting on sex and gender	N/A
Reporting on race, ethnicity, or other socially relevant groupings	N/A
Population characteristics	N/A
Recruitment	N/A
Ethics oversight	N/A

Note that full information on the approval of the study protocol must also be provided in the manuscript.

Field-specific reporting

Please select the one below that is the best fit for your research. If you are not sure, read the appropriate sections before making your selection.

☒ Life sciences ☐ Behavioural & social sciences ☐ Ecological, evolutionary & environmental sciences

For a reference copy of the document with all sections, see nature.com/documents/nr-reporting-summary-flat.pdf

Life sciences study design

All studies must disclose on these points even when the disclosure is negative.

Sample size

Sample sizes were guided by prior experience and published literature. No statistical methods were used to pre-determine sample size or effect size. Time-lapse U2OS cell experiments were performed with 2–5 biological replicates, with key experiments repeated in at least three independent replicates to ensure reproducibility. Single time-point U2OS cell assays (Fig. 1a (right panel), 2c, Extended Data Fig.1a, Extended Data fig. 2g, and Extended Data Fig. 2h) were conducted in one independent experiment performed on a single day, using cells from two independent frozen vials. Equilibrium GUV experiments (extended data Fig. 2a) were performed as a single independent experiment on a single day, using GUVs from two independently prepared lipid mixtures. For zebrafish imaging, each experiment represents a biologically independent replicate using embryos derived from distinct clutches. Experiments were repeated across multiple days, with one embryo imaged per experiment for each UV- or IR-induced tissue wounding condition, and repeated on more than six different live embryos to ensure reproducibility.

Data exclusions

Data exclusion criteria were predefined. Imaging datasets were excluded if affected by artificial fluorescence fluctuations (e.g., immersion oil bubbles), incompletely acquired Z-stacks or broken tracks were either cropped or completely discarded. All raw imaging files of cell experiments were loaded into custom written automatic programs for analysis, unless otherwise stated. For the nuclear membrane analysis program, segmented objects with size less than 8000 pixel2 (~140 um^2) and/or touching the borders of the image boundary were excluded from the analysis. Nuclei were removed from analysis If when segmented objects moved more than 130 pixel (~20 µm) between successive frames or broken tracks appeared due to leakage of nucleoplasmic fluorescent markers (cPla2-mKate2, ALPIN) when these markers were used for nuclear segmentation, these nuclei were removed from analysis. Multiple tracks that were detected as single were deleted from the analysis. For the ER analysis program, segmented ER vesicles size less than 30-50 pixel2 (~ 1 µm2) were precluded from analysis. Since the ER was analyzed per FOV, no tracks were involved, and all time points were included in the analysis unless ER segmentation was precluded by the leakage of eGFP-KDEL. In long-term cell imaging experiments, datasets were excluded only when imaging artifacts prevented reliable analysis and data extraction. Specifically, exclusions were made if (i) after fluorescent markers leaked from their designated organelle localization into the surrounding cytoplasmic space during acquisition, (ii) cells became clumped, or (iii) automated tracking produced broken or fragmented tracks. For GUV experiments, the raw imaging files were loaded into a semi-automatic custom written analysis program where individual GUV was randomly selected by the user for analysis. For zebrafish live embryo imaging, datasets were excluded if tailfin tissue drift exceeded 100

	pixels (~50 µm) in the XY plane or 20 pixels (~10 µm) in the Z-direction following UV- or IR- laser damage. All other biologically independent replicates were retained for statistical analyses, and no additional outliers were excluded.
Replication	Typically, experiments were repeated at least on two separate days (using cells thawed from different vials). Only the experiments shown in Fig. 1a (right panel), 2c, Extended Data fig.1a, Extended Data fig.2a, Extended Data Fig. 2g, and Extended Data fig. 2h were not replicated on different days, yet nevertheless conducted using cells or GUVs prepared from at least two independent stock sources, i.e., storage vials or lipid mixtures, respectively. For zebrafish embryos imaging experiments were repeated on two separate days using independent clutch of embryos. Statistical analysis were performed across biological replicates. Replication included either variation in biological source material and/or independent imaging sessions conducted on separate experimental days.
Randomization	Selection of animal larvae and cells for experiments is randomized without predetermined grouping, bias or selection.
Blinding	Analysis was performed objectively without blinding. Data processing and quantification were carried out using an automated computational pipeline, with no manual intervention or subjective data selection prior to analysis.

Reporting for specific materials, systems and methods

We require information from authors about some types of materials, experimental systems and methods used in many studies. Here, indicate whether each material, system or method listed is relevant to your study. If you are not sure if a list item applies to your research, read the appropriate section before selecting a response.

Materials & experimental systems

n/a	Involved in the study
☒	☐ Antibodies
☐	☒ Eukaryotic cell lines
☒	☐ Palaeontology and archaeology
☐	☒ Animals and other organisms
☒	☐ Clinical data
☒	☐ Dual use research of concern
☒	☐ Plants

Methods

n/a	Involved in the study
☒	☐ ChIP-seq
☒	☐ Flow cytometry
☒	☐ MRI-based neuroimaging

Eukaryotic cell lines

Policy information about cell lines and Sex and Gender in Research

Cell line source(s)	ATCC
Authentication	Since cell lines were obtained from commercial sources, no authentications were performed.
Mycoplasma contamination	Cell lines were not tested for Mycoplasma contamination
Commonly misidentified lines (See ICLAC register)	No commonly misidentified lines were used in the study

Animals and other research organisms

Policy information about studies involving animals; ARRIVE guidelines recommended for reporting animal research, and Sex and Gender in Research

Laboratory animals	Adult casper zebrafish were reared in 2.8-liter polycarbonate tanks at a density of 10 fish per liter. Fish were maintained in salinity-conditioned system water at 28 °C under a 14:10-hour light:dark photoperiod cycle.
Wild animals	Study did not involve any wild animals.
Reporting on sex	Sex are indeterminate in the larval stage at which the presented experiments are conducted.
Field-collected samples	The study did not involve samples that were directly collected from the field.
Ethics oversight	Experiments are conducted according to institutional animal healthcare guidelines with the approval of the Institutional Animal Care and Use Committee (IACUC). MSKCC Protocol Number:11-01-002

Note that full information on the approval of the study protocol must also be provided in the manuscript.

Plants

Seed stocks	N/A
Novel plant genotypes	N/A
Authentication	N/A
