# Supplementary figures and images for: Endoplasmic reticulum disruption stimulates nuclear membrane mechanotransduction
Source: Nat Cell Biol. 2025 Dec 9;28(1):125–34. doi: 10.1038/s41556-025-01820-9 (PMC12807876; doi:10.1038/s41556-025-01820-9)

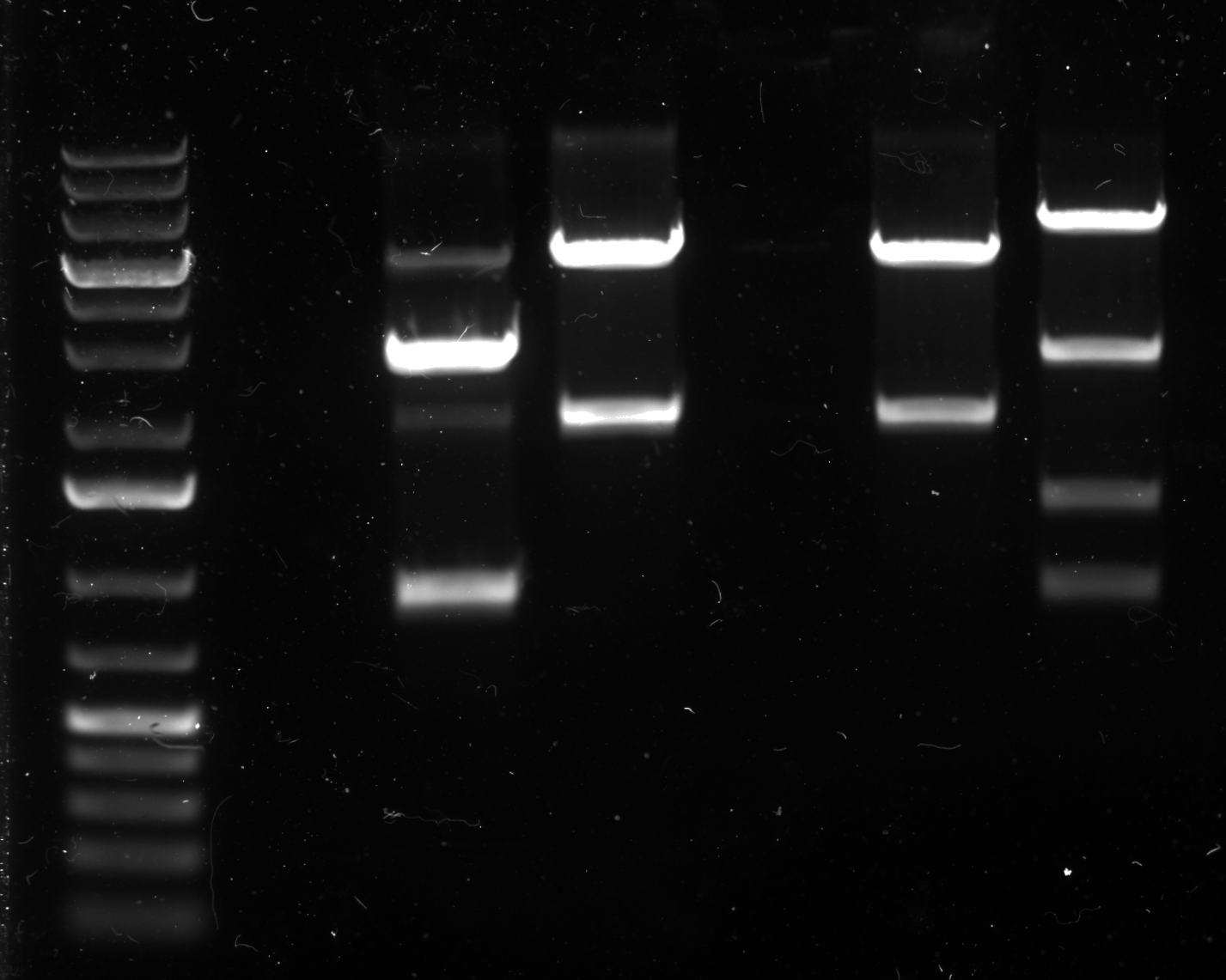

Supplement: Supplementary file 18 — Unprocessed DNA gel showing restriction enzyme digest fragment patterns supporting Extended Data Fig. 5b. [file 41556_2025_1820_MOESM18_ESM.tif]
